# BRAF Mutant Gastrointestinal Stromal Tumor: First report of regression with BRAF inhibitor dabrafenib (GSK2118436) and whole exomic sequencing for analysis of acquired resistance

**DOI:** 10.18632/oncotarget.864

**Published:** 2013-02-25

**Authors:** G.S. Falchook, J.C. Trent, M.C Heinrich, C. Beadling, J. Patterson, C.C. Bastida, S.C. Blackman, R. Kurzrock

**Affiliations:** ^1^ Department of Investigational Cancer Therapeutics (Phase I Clinical Trials Program), Division of Cancer Medicine, The University of Texas M.D. Anderson Cancer Center, Houston, Texas; ^2^ Sarcoma Medical Oncology Program, University of Miami, Sylvester Comprehensive Cancer Center, Miami, Florida; ^3^ Portland VA Medical Center and Oregon Health & Science University (OHSU) Knight Cancer Institute, Division of Hematology & Oncology, Portland, Oregon; ^4^ GlaxoSmithKline, Collegeville, PA; ^5^ Moores Cancer Center, Department of Medicine, Hematology-Oncology Division, The University of California, San Diego, La Jolla, CA

**Keywords:** Gastrointestinal stromal tumor, Dabrafenib, GSK2118436, BRAF mutation, BRAF inhibition, V600E

## Abstract

Activating oncogenic mutations of BRAF have been described in patients with gastrointestinal stromal tumor (GIST), but treatment of GIST with BRAF inhibitors and mechanisms of mediating the emergence of resistance in GIST have not been reported. Dabrafenib is a potent ATP-competitive inhibitor of BRAF kinase and is highly selective for mutant BRAF in kinase panel screening, cell lines, and xenografts. We report prolonged antitumor activity in the first patient with V600E BRAF-mutated GIST who was treated with a BRAF inhibitor. Whole exome sequencing performed in tumor tissue obtained at the time of progressive disease demonstrated a somatic gain-of-function PIK3CA mutation (H1047R) as well as a CDKN2A aberration, which may have contributed to eventual resistance to treatment.

## INTRODUCTION

Gastrointestinal stromal tumor (GIST) is a malignancy of mesenchymal origin that arises in the gastrointestinal tract and is resistant to conventional cytotoxic chemotherapy agents[1]. KIT and platelet-derived growth factor receptor-α (PDGFRA) mutations are present in 80% and 8% of GISTs, respectively[2-4]. Approximately 13% of KIT and PDGFRA wild-type GISTs contain BRAF mutations[5]. Although receptor tyrosine kinase inhibitors, such as imatinib or sunitinib, are therapeutically active antagonists of KIT and PDGFRA in KIT- or PDGFRA-mutated GIST[6-8], effective treatments for patients with advanced BRAF-mutant GIST have not been reported.

Clinical trials of tyrosine kinase inhibitors that are highly selective for V600 BRAF mutations have demonstrated high response rates (50-80%) in BRAF-mutant melanoma, as well as improvement in overall survival and progression-free survival[9-11]. Recently, we have shown that the BRAF inhibitor dabrafenib (GSK2118436) is also active in several non-melanoma BRAF-mutated cancers[10].

Herein, we report antitumor activity in the first patient with BRAF-mutated GIST who was treated with a BRAF inhibitor. Whole exome sequencing of tumor obtained at time of progressive disease did not reveal secondary BRAF or RAS mutations, but did demonstrate a somatic gain-of-function PIK3CA mutation (H1047R) as well as a CDKN2A aberration, which may have been responsible for dabrafenib resistance.

## RESULTS

A 60 year old man initially presented in September 2007 with abdominal pain and a palpable mass. Computed tomography (CT) revealed a 10 cm heterogeneous mass, and a subsequent biopsy demonstrated GIST, spindled cell histology, positive for CD34 and CD117 by immunohistochemistry with 6 mitoses per 10 high-powered fields. The patient underwent surgical resection revealing a 15 cm mass. DNA was extracted from formalin-fixed paraffin-embedded tumor tissue and subjected to polymerase chain reaction (PCR) amplifications of KIT exons 9, 11, 13, and 17 as well as PDGFRA exons 12 and 18. Sanger sequencing did not identify mutations in either the KIT or PDGFRA genes. The patient presented with a new 14 cm mass at the dome of the bladder after 10 months of adjuvant imatinib therapy (400 mg once daily). The imatinib dose was increased to 800 mg daily, followed by surgical resection of the mass. The patient received adjuvant sunitinib, a multiple tyrosine kinase inhibitor, at a dose of 50 mg on a schedule of once daily for four weeks, then off for two weeks. Nineteen months later, a PET/CT showed recurrent FDG-avid masses in the right internal iliac region and in the right abdomen extending into the rectus abdominis.

The patient enrolled on a clinical trial with an investigational KIT/PDGFRA/VEGFR tyrosine kinase inhibitor, but disease progression was noted at his first restaging (two months of treatment). Further testing of the patient’s original tumor revealed a V600E BRAF mutation. The patient was then treated with an investigational MEK inhibitor for three months, during which the tumor initially remained stable but was subsequently found to have enlarged and remained enhancing by CT imaging.

**Figure 1:**
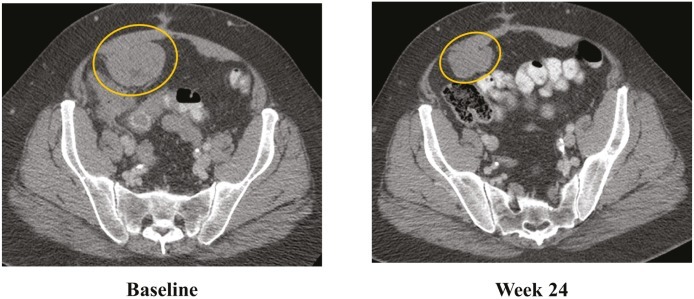
Tumor regression of 20% observed in abdominal and pelvic tumors on computerized tomography (CT) CT scan at (a) baseline and after (b) 24 weeks of treatment with BRAF inhibitor dabrafenib (GSK2118436).

The patient was treated on a phase I trial of dabrafenib at a dose of 150 mg twice daily[10]. The patient’s baseline CT scan demonstrated multiple metastases in the lower abdomen and pelvis, with the largest tumors including a 6.3 cm mass posterior to the bladder and a 6.3 cm mass in the anterior pelvis (Figure 1, Panel A). Using the Response Evaluation Criteria in Solid Tumors (RECIST) 1.0, restaging scans revealed a 14%, 18% and 20% decrease after 6, 15 and 24 weeks of treatment, respectively. Figure 1 Panel B demonstrates response on CT scan at 24 weeks. In addition, the tumor demonstrated a marked decrease in contrast enhancement, a response criteria that has been validated in GIST[12].

The patient remained on study for 8 months, after which tumor progression was noted by contrast-enhanced CT imaging. The only treatment-related adverse events were grade 2 rash and acrochrodons (skin tags), as well as grade 1 fatigue and hyperkeratosis of the plantar surface of the feet. After tumor progression was identified, the patient underwent surgical resection of all visible tumors in the abdomen and pelvis. Tissue from this resection was evaluated with whole exome sequencing.

**Figure 2:**
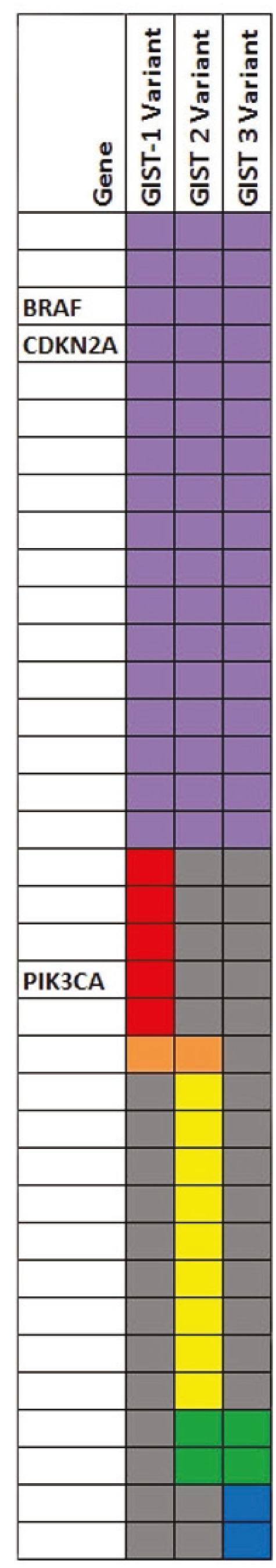
Three tumors were analyzed by whole exome sequencing

**Figure 3:**
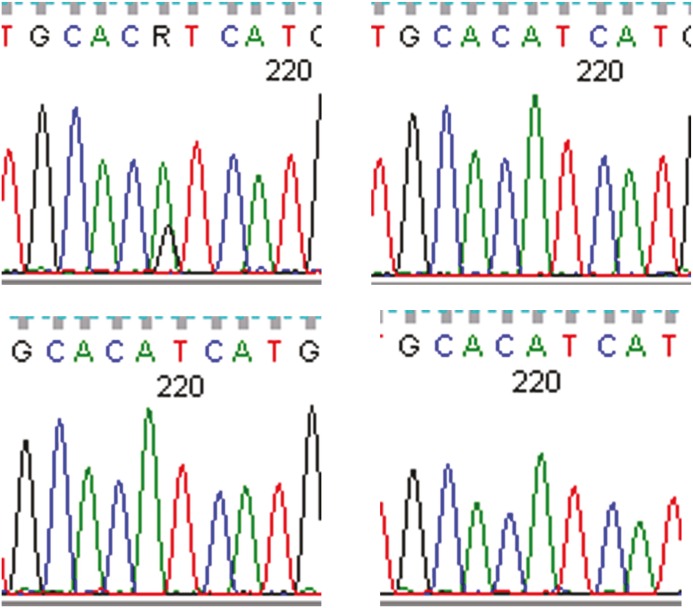
Lesion 1 (a) had a gain of function PIK3CA H1047R mutation while lesion 2 (b), lesion 3 (c), and normal tissue (d) were wild type for PIK3CA

To fully account for intratumor heterogeneity, which can be a factor in tumor adaptation and treatment failure[13], three lesions were analyzed by whole exome sequencing (Figure 2). All three lesions were clonally related as evidenced by identical BRAF V600E mutations, identical CDKN2A IVS1+1 G>A mutations, and fifteen other shared somatic single nucleotide variations. One of the three lesions (lesion 1), had a somatic gain-of-function PIK3CA mutation (H1047R), that has previously been reported in other human cancers[14]. Figure 3 demonstrates the PIK3CA H1047R mutation in lesion 1 (Panel A), in contrast to wild type PIK3CA in lesion 2 (Panel B), lesion 3 (Panel C), and normal tissue (Panel D). Lesions 2 and 3 appeared to be clonally related as they shared two mutations that were not present in lesion 1. Although all three lesions had a common CDKN2A mutation, lesions 1 and 3 were heterozygous for this mutation whereas lesion 2 was homozygous. This splice site mutation has been described previously as a somatic variant in melanoma[15] and glioma[16].

## DISCUSSION

BRAF inhibitors have demonstrated antitumor activity in clinical trials of patients with BRAF mutant malignancies[9-11]. We report prolonged antitumor activity in the first patient with a BRAF-mutated GIST who was treated with a BRAF inhibitor.

Activating oncogenic mutations of BRAF have been described in many malignancies, including cutaneous melanoma (67%), colorectal carcinoma (12%), non-small cell lung carcinoma (NSCLC; 3%), and KIT wild-type GIST (13%)[5,17]. The most common BRAF mutation is a substitution of valine with glutamic acid at amino acid position 600 (V600E), which locks BRAF into its active conformation, resulting in a ten-fold increase in activity over wild-type BRAF[17].

Dabrafenib is a potent ATP-competitive inhibitor of BRAF kinase and is highly selective for mutant BRAF in kinase panel screening, cell lines, and xenografts[18]. Dabrafenib has demonstrated antitumor activity in several BRAF-mutated malignancies including melanoma, colorectal carcinoma, papillary thyroid carcinoma, NSCLC, and ovarian carcinoma[10].

Kinase inhibitors targeting BRAF have the potential to be an effective therapeutic option for BRAF-mutant GIST patients[10]. The present case demonstrates proof of principle for BRAF inhibition as a therapeutic strategy for GIST patients. Tumor regression was not seen when this patient was given a multi-kinase inhibitor that did not target BRAF, or a MEK inhibitor. However, it should be noted that both of these agents were experimental, and therefore their therapeutic value has not yet been fully validated. Treatment with dabrafenib, which targets BRAF directly, resulted in tumor regression after 6 weeks, and continued decreasing in size until week 24, followed by a plateau and then progression at 8 months.

Whole exome sequencing did not reveal secondary BRAF or RAS mutations but did demonstrate a somatic gain-of-function PIK3CA mutation (H1047R), that has previously been reported in other human cancers[14]. We speculate that the PIK3CA mutation could be the cause of the acquired BRAF inhibitor resistance in lesion 1. This finding is notable, because to the best of our knowledge this is only the second PIK3CA mutation ever reported in GIST[19]. Furthermore, although PIK3CA mutations have not previously been reported as a cause of acquired resistance to BRAF inhibitors in melanoma or other malignancies, low PTEN expression and other PTEN alterations are associated with lower response rate and shorter progression-free survival in BRAF mutant melanoma patients treated with BRAF inhibitors[20,21]. We further speculate that dysregulation of cell cycle control by the homozygous CDKN2A mutation in lesion 2 may also be a molecular basis for resistance of this lesion. No obvious explanation for resistance to BRAF inhibitor treatment was seen in lesion 3. We further tested RNA from all three lesions and were unable to detect aberrant BRAF splicing as a basis for drug resistance[22]. The differences in sequencing among the three lesions highlight the prevalence of intratumor heterogeneity and the potential relevance to treatment outcomes[13].

In conclusion, we present the first patient with GIST and a V600E BRAF mutation whose tumor showed regression while receiving treatment with a BRAF inhibitor. To our knowledge, the efficacy of BRAF inhibitors in BRAF-mutant GIST has not been reported, but our case suggests that additional studies and perhaps a global clinical trial are warranted.

## MATERIALS AND METHODS

Whole exome capture was performed with a SeqCap EZ Human Exome v2.0 kit (Roche NimbleGen, Madison, WI), and sequencing was carried out on a HiSeq 2000 instrument (Illumina Inc, San Diego, CA). Sequence alignment and variant calling were performed with DNAnexus software (DNAnexus Inc, Mountain View, CA). Tumor-specific variants were identified based on a minimum variant allele ratio of 20%, a minimum read depth of 20, and absence of the variant in a matched normal specimen. Nucleotide variants were translated, and non-synonymous variants were identified using SIFT[23], PolyPhen2[24], and Mutation Assessor[25]. Variants of interest were confirmed by Sanger sequence analysis.
